# On Demand Secure Scalable Video Streaming for Both Human and Machine Applications

**DOI:** 10.3390/s26041285

**Published:** 2026-02-16

**Authors:** Alaa Zain, Yibo Fan, Jinjia Zhou

**Affiliations:** 1 Faculty of Science and Engineering, Department of Applied Informatics, Koganei Campus, Hosei University, Tokyo 102-8160, Japan; 2 Fudan University, Shanghai 200437, China

**Keywords:** deep video coding, video coding for machines, scalable video coding, encrypted video streaming, scalable video streaming

## Abstract

Scalable video coding plays an essential role in supporting heterogeneous devices, network conditions, and application requirements in modern video streaming systems. However, most existing scalable coding approaches primarily optimize human perceptual quality and provide limited support for data privacy, as well as for machine analyses and the integration of heterogeneous sensor data. This limitation motivated the development of adaptive scalable video coding frameworks. The proposed approach is designed to serve both human viewers and automated analysis systems while ensuring high security and compression efficiency. The method adaptively encrypts selected layers during transmission to protect sensitive content without degrading decoding or analysis performance. Experimental evaluations on benchmark datasets demonstrate that the proposed framework achieves superior rate distortion efficiency and reconstruction quality, while also improving machine analysis accuracy compared to existing traditional and learning-based codes. In video surveillance scenarios, where the base layer is preserved for analysis, the proposed scalable human machine coding (SHMC) method outperforms scalable extensions of H.265/High Efficiency Video Coding (HEVC), Scalable High Efficiency Video Coding (SHVC), reducing the average bit-per-pixel (bpp) by 26.38%, 30.76%, and 60.29% at equivalent mean Average Precision (mAP), Peak Signal-to-Noise Ratio (PSNR), and Multi-Scale Structural Similarity (MS-SSIM) levels. These results confirm the effectiveness of integrating scalable video coding with intelligent encryption for secure and efficient video transmission.

## 1. Introduction

Video streaming is an essential technology with applications across diverse domains, including visual surveillance, traffic control, autonomous navigation video conferencing, and digital broadcasting [1]. In many cases, video input is first compressed then sent to the cloud for further analysis. The approach taken often depends on the target application. For systems focused solely on machine-based video analysis, it is possible to transmit compressed precomputed features instead of the full video, which significantly reduces bandwidth requirements [2,3]. Recently both traditional hand-crafted methods and modern neural network-based techniques have been explored to achive this [4,5]. On the other hand, when human viewing is also required, the system must encode and transmit the original video, thereby increasing the overall complexity.

The rapid development of deep learning has introduced a new trend developing learned compression frameworks for images and videos using deep neural networks (DNNs). These models attempt to advance traditional coding standards such as JPEG, HEVC, and VVC [6,7,8,9,10]. The majority of DNN-based compression techniques have been primarily optimized for human visual perception and not necessarily machine-focused analysis. While deep networks are widely used in visual perception and understanding problems, compression itself has seldom been the exclusive focus of such models [9].

To bridge the gap, ref. [11] introduced JPEG AI, a deep learning-based image standard coder that integrates human visual viewing and machine vision into a single bitstream. One significant benefit of JPEG AI over its previous versions is that it accommodates direct input of entropy decoded latent features into analytics based on DNN without the necessity of complete reconstruction of the image, thereby reducing computational cost. Nonetheless, the standard lacks the ability to separate task related from task unrelated content at the encoding stage. Therefore, in use cases where human eye observation is not common, JPEG AI is nevertheless not optimally bit efficient because its bitstream contains information undesirable for machine vision applications.

In order to go beyond these constraints, new standardization work initiatives such as MPEG-VCM (Video Coding for Machines) and MPEG-FCM (Feature Coding for Machines) have been lunched. These are aimed at defining harmonized frameworks for both catering human vision and machine vision. At the same time, several scalable image coding techniques for multi-tasking tasks have been introduced [12,13,14]. They are normally in the format of layer-based where the lowest layer enables machine-centric analysis (e.g., tracking or object detection), and enhancement layers on top enable reconstruction for human visibility.

In video streaming, internet-delivered services have been supported by digital rights management (DRM) systems that manage copyrights and restrict access in a way that is controlled. Encryption is the primary activity of DRM to stop the unauthorized sharing of copyrighted video streams. End-to-end encryption helps ensure that only legitimate users are able to decrypt and watch the delivered content.

**Figure 1:**
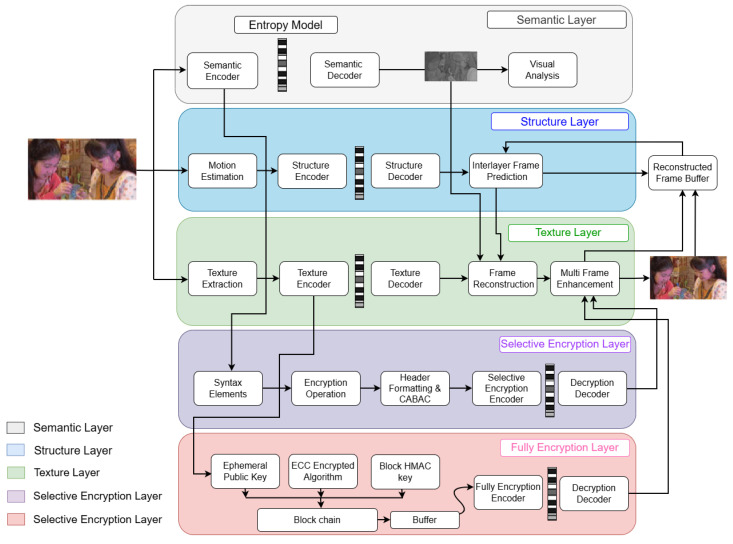
Overview of the proposed adaptive SVHM framework. The input sequence presented in the figure is selected from the standard HEVC common test sequences [48].

Recently, blockchain-inclined encryption schemes have been explored to enhance transparency and processing efficiency in secure video streaming. However, the majority of them are plagued with performance deterioration over unencrypted streaming. To address these issues, we present an adaptive scalable human–machine video coding (HMSVC) framework for encrypted video streaming applications. The presented approach degrades every video into a number of quality layers, as depicted in Figure 1. Every layer has a specific resolution or degree of fidelity, which can be utilized to adjust transmission based on available bandwidth or capability of the receiver device. The video conferencing server selects dynamically decodes layers best suited for current network conditions and user requirements. Such a scheme ensures a seamless play and uniform visual look even with varying network constraints.

The major contributions presented in this paper are summarized as follows:

(1) We introduce a new adaptive, scalable encrypted system combining blockchain and region-of-interest (ROI)-based encoding for centralized video streaming usage. To our best knowledge, it is the first adaptation of adaptive blockchain-based scalable streaming for ensuring video integrity in such environments.
(2) To address the scalability problems of blockchain with large transactional loads, our design further includes a user-manageable feature for prioritizing critical videos to ensure safe delivery. The system can dynamically scale to meet the requirements of different video applications.
(3) The proposed ROI mechanism reduces computational overhead and memory usage in integrity verification, thereby allowing for more rapid operation.
(4) We conducted extensive experiments analysis of possible security risks and attacks and show that the suggested integrity verification framework is very secure and robust.

The remainder of this paper is organized as follows. Section 2 presents the related works. Section 3 describes the proposed methods experimental setup and evaluation metrics. Section 4 shows the experimental configurations. Section 5 discusses the experimental results and performance comparisons. Finally, Section 6 concludes the paper and outlines future research directions.

## 2. Related Works

### 2.1. Video Coding for Human Vision

Well-known standards video coding such as H.264/AVC [15], H.265/HEVC [16,17], and H.266/VVC [18] have largely been designed to remove spatial and temporal redundancies in order to compress efficiently [19]. These standards leverage well-designed modules, which have been made highly efficient in performance over time. Deep learning, which has emerged in recent times, has encouraged a new generation of neural network-based codecs to learn compressing video content end-to-end [20].

Recent research, for instance [21], replaced conventional modules such as motion estimation and compensation with joint learning neural networks to achieve optimal end-to-end rate distortion performance. This research is furthered by Wang et al. [22], who presented a flow-guided feature prediction module to facilitate better feature alignment and designed a temporal context compression module to replace residual coding to better exploit inter-frame redundancy. Rippel et al. [23] introduced an adaptive rate control framework, while Li et al. [5] came up with conditional coding leveraging temporal correlations to better compress current frames. Follow-up research [24,25,26,27,28] further enhanced efficiency in compression through feature propagation and multi-scale temporal backgrounds.

Although these learning-based codecs achieve superb perceptual quality, their preference is still designed according to human vision experience. This harmony could undermine their efficiency in bandwidth-constrained settings or when videos are read predominantly by machine systems [29]. To bridge the gap, we introduce a CSC-l network, which expresses video features in a form well suited for machine understanding, achieving an even better bitrate vs. analytical precision trade off.

### 2.2. Video Coding for Machine Analysis

Unlike codecs for human perception, coding research based on machines emphasizes more task-oriented feature extraction and compression rather than pixel reconstruction. MPEG standardized Compact Descriptors for Visual Search (CDVS) [30] and Compact Descriptors for Video Analysis (CDVA) [31] at an early stage, which established compact representations for recognition and retrieval. Multi-modal feature fusion has shown strong effectiveness in semantic video understanding tasks such as dense video captioning [32,33,34]. However, its adoption in efficient video compression and transmission frameworks remains limited. Next, Chen et al. [35] explored the encoding of middle level deep features using traditional codecs, while more recent work [11,36,37,38,39,40] pursued learning-based strategies that optimize both compression and the analysis task outcome in a concurrent manner. However, one intrinsic challenge still persists balancing efficient feature compression for robust machine inference with the capability to reconstruct high quality video for human vision.

### 2.3. Unified Video Coding for Human and Machine Vision

New advances have attempted to balance human inspection goals with machine analysis objectives. Some approaches use a shared bitstream in common [35], training neural codecs with task specific loss functions enabling the reconstructed video to support both visual quality and analytical performance at the same time. Yang et al. [41] developed this concept with learned semantic representations encoding motion- and object-level information for machines but enabling human visual reconstruction.

Alternatively, scalable coding techniques [42,43,44] use multiple layers: a dense base layer that contains task specific features to be processed by machines, and refinement layers that reconstruct visual content to be perceived by humans. For example, Wang et al. [42] and Hu et al. [12] designed scalable frameworks for facial image analysis, and Choi et al. [14] divided latent features into independent subspaces to accommodate multiple tasks in an integrated model.

Despite these advancements, the majority of existing approaches are not optimized for video data that is temporal [13,14,45], limiting their performance when directly applied to video data. Straightforward application to video analysis will likely generate larger bitrates as well as suboptimal general compression efficiency.

### 2.4. Video Streaming and Secure Transmission

With the rise in the number of streaming services at a fast rate, data secrecy during transmission has turned out to be a fundamental requirement. Recent advances in intelligent sensing systems increasingly rely on the integration of artificial intelligence with cloud and edge computing to support efficient data processing and transmission across distributed environments. Such architectures are particularly relevant for secure video streaming, where large volumes of visual data must be handled with low latency and reliability [46]. At the communication level, emerging 6G networks are expected to further enhance secure and intelligent video delivery by incorporating AI-driven mechanisms for network management, security, and adaptive resource allocation [47]. Despite these developments, ensuring efficient, secure, and scalable video streaming remains a challenging problem, especially for high-resolution and real-time video content. Modern systems couple encryption mechanisms such as the Advanced Encryption Standard (AES), Data Encryption Standard (DES), Rivest Shamir Adleman (RSA), and Searchable Encryption (SE) with video compression standards such as MPEG and HEVC. These hybrid systems attempt to meet high efficiency as well as robust security for digital content during internet delivery.

## 3. Proposed Method

In this section, the proposed Scalable Human–Machine Coding (SHMC) framework is presented. First, the adaptive layer selection mechanism and then the joint optimization problem of human and machine vision are explained. After that, there is a discussion about framework overview, and selective and full encryption strategies.

### 3.1. Adaptive Selection and Optimization Formulation

To deal with the unique requirements of machine and human vision, video coding is modeled as a four branch optimization problem. The first branch, which is the machine branch, optimizes for feature distortion minimization given a bitrate constraint. Once this branch is optimized, the human branch is optimized to reconstruct high-quality video and with its own bitrate constraint.

This can be modeled mathematically as

(1) $$\theta_{m}^{*}=\arg\min_{\theta_{m}}D_{m},\;\mathrm{subject}\;\mathrm{to}\;R_{m}<R_{c}^{m},$$

(2) $$\theta_{h}^{*}=\arg\min_{\theta_{h}|\theta_{m}^{*}}D_{h},\;\mathrm{subject}\;\mathrm{to}\;R_{h}<R_{c}^{h},$$

where $\theta_{m}$ and $\theta_{h}$ denote the coding parameters for the machine and human branches, respectively; $D_{m}$ and $D_{h}$ represent the corresponding distortion metrics; and $R_{m}$, $R_{h}$, $R_{c}^{m}$, and $R_{c}^{h}$ are the actual and maximum allowed bitrates for each branch. This formulation explicitly models the dependency of the human branch on the optimized machine parameters, ensuring efficient performance for both machine analysis and human visual reconstruction.

The proposed framework is shown in Figure 1 integrates five layers semantic, structure, texture, selective encryption, and fully encryption layers for efficient secured video compression and reconstruction. The application selection can be implemented using the user interface choice.

Interface layer selection:

- u∈{1,2,3,4} denotes the user branch choice:
  – 1: human
  – 2: machine
  – 3: selective encryption
  – 4: fully encrypted
- $L=[L_{1},L_{2},L_{3},L_{4},L_{5}]$ is the layer activation vector, where
  $$L_{i}=\left\{\begin{array}{cc} 1, & \mathrm{if}\;\mathrm{layer}\;i\;\mathrm{is}\;\mathrm{activated}\;\mathrm{for}\;\mathrm{the}\;\mathrm{chosen}\;\mathrm{branch},\\ 0, & \mathrm{otherwise}. \end{array}\right.$$

(3) $$L\left(u\right)=\left\{\begin{array}{cc} {}[0,1,1,0,0], & u=1\;(\mathrm{human})\\ {}[1,0,1,0,0],, & u=2\;(\mathrm{machine})\\ {}[1,0,1,1,0],, & u=3\;(\mathrm{selective}\;\mathrm{encryption})\\ {}[0,1,1,0,1],, & u=4\;(\mathrm{fully}\;\mathrm{encrypted}) \end{array}\right.$$

### 3.2. Framework Overview

As shown in Figure 1, the resulting SHMC framework consists of five functional layers: semantic layer, structure layer, texture layer, selective encryption layer, and full encryption layer.

These layers cooperate to provide efficient, scalable, and secure compression and reconstruction of the video. The system also features a user interface that allows for dynamic choice of the target branch based on application needs.

### 3.3. Selection of Interface Layer

Based on the activation components defined in (3), the operating state of each branch is represented using a unified activation vector L(u). Each element of this vector corresponds to a specific processing layer, namely the semantic, structural, texture, selective encryption, and full encryption layers. This representation provides a consistent notation and explicitly describes how content characteristics determine the active processing and encryption strategy.

(4) $$L\left(u\right)=\left\{\begin{array}{cc} {}[0,1,1,0,0], & u=1\;(\mathrm{human-oriented}\;\mathrm{branch})\\ {}[1,0,1,0,0],, & u=2\;(\mathrm{machine-oriented}\;\mathrm{branch})\\ {}[1,0,1,1,0],, & u=3\;(\mathrm{selective}\;\mathrm{encryption}\;\mathrm{branch})\\ {}[0,1,1,0,1],, & u=4\;(\mathrm{full}\;\mathrm{encryption}\;\mathrm{branch}) \end{array}\right.$$

The first three elements of L(u) indicate the activation of the semantic, structural, and texture layers, while the last two elements specify the applied encryption mode. Depending on the semantic sensitivity of the content, either selective encryption or full encryption is enabled, ensuring that only one encryption strategy is active for a given branch.

Therefore, the active configuration for any branch is specified as Algorithm 1.

The GUI for interface selection enabling users to select adaptively between branches according to the application context and security requirements.

### 3.4. Semantic Layer

The semantic layer employs a Conditional Semantic Compression (CSC) network, drawing inspiration from the [49] architecture, to compress high level semantic features from consecutive frames for machine understanding. Segmentation sequences preserve geometric structure by maintaining object boundaries and spatial relationships between frames. The optimization objective is as per Equation (1).

The structure layer also records motion-based structural information through a motion estimation module. The lower quality frames are predicted using an Interlayer Frame Prediction (IFP) network, optimized using Equation (2), while the motion information is propagated efficiently. The texture residuals are also enhanced through a U Net-based reconstruction module, which maintains visual details by optimizing perceptual quality estimation (PQE).

### 3.5. Texture Layer

The texture layer focuses on generating high fidelity video suitable for human vision [49]. All the compression modules use a channel-wise auto-regressive (CAR) entropy model to compress quantized features into bitstreams.
**Algorithm 1** Branch-based layer selection with adaptive intrusion detection**Require:** 
Branch type u∈{human,machine,selective_encryption,fully_encrypted}**Require:** 
Intrusion flag intrusion_detected∈{0,1}**Ensure:** 
Activated layer list 1:activated_layers←[] 2:**if** intrusion_detected=1 **then** 3:    activated_layers←[2,3,5]                                                ▹ Force full encryption 4:**else** 5:    **if** u=human **then** 6:        activated_layers←[2,3] 7:    **else if** u=machine **then** 8:        activated_layers←[1,3] 9:    **else if** u=selective_encryption **then**10:        activated_layers←[1,3,4]11:    **else if** u=fully_encrypted **then**12:        activated_layers←[2,3,5]13:    **end if**14:**end if**15:**return** activated_layers

When selective encryption is turned on, the system selectively encrypts significant regions of every frame. The video stream is broken down into Coding Tree Units (CTUs), and the encoding process continues with normal procedures like intra/inter prediction, deblocking, Sample Adaptive Offset (SAO), transform, quantization, and entropy coding.

### 3.6. Selective Encryption Layer

Entropy coding is the final stage of the compression pipeline and is responsible for making data lossless by eliminating statistical redundancy in the syntax structures. In the High Efficiency Video Coding (HEVC) standard, this is done through Context-based Adaptive Binary Arithmetic Coding (CABAC).

CABAC is implemented in two modes: regular and bypass. In the normal mode, each binary symbol’s probability is determined according to the context established by symbols already encoded. Such a probability model is updated step by step as the encoding continues, and then a binary arithmetic encoder is used to generate the compressed bitstream. If encryption is applied in this mode, it may subtly alter the probability model so that there are minor variations in the overall bitstream size. Algorithms 2 and 3 show the steps of encryption and decryption in the intrusion scenario.

In bypass mode, all the syntax elements are treated as uniformly likely and separately encoded, resulting in faster processing. In comparison to the regular mode, encryption in bypass mode has no effect on compression efficiency or changes the bitstream probability model.
**Algorithm 2** Selective encryption scheme**Require:** 
Raw video *V*, secret key *K*, ROI map based on semantic layer**Ensure:** 
Encrypted HEVC/VVC-compliant bitstream 1:Extract frames $\left\{F_{t}\right\}$ from *V* 2:Detect sensitive objects and generate ROI masks 3:**for** each Coding Tree Unit (CTU) **do** 4:    Identify syntax elements associated with ROI 5:    Select bypass-coded elements (QTC, MVD, optional IPM) 6:    **for** each selected syntax element **do** 7:        Perform CABAC binarisation to obtain bin sequence $P=\{P_{i}\}$ 8:        Obtain current Most Probable Symbol (MPS) 9:        **for** each bin $P_{i}$ **do**10:           ${\tilde{P}}_{i}\leftarrow P_{i}⊕\mathrm{MPS}$11:           $S_{i}\leftarrow {\mathrm{AES}}_{128}\left(C_{i-1},K\right)$12:           $C_{i}\leftarrow S_{i}⊕{\tilde{P}}_{i}$13:           Send $C_{i}$ directly to arithmetic encoder14:        **end for**15:        **if** syntax element is non-binary (QTC or MVD) **then**16:           Scramble magnitude using Linear Congruential function17:           $X_{i}^{\prime }\leftarrow \left(aX_{i}+b\right)\;\mathrm{mod}\;m$18:        **end if**19:    **end for**20:**end for**21:Assemble arithmetic-coded bins into encrypted bitstream22:**return** Encrypted bitstream

SE is applied prior to CABAC is entered to ensure content protection without format compliance compromise. The processing typically involves three main steps:

Step 1: Encryption Target Selection. The encryption begins with identifying what the syntax components to be encrypted. These must be within the HEVC framework to avoid format infringements. More often than not, Quantized Transform Coefficients (QTC) and Motion Vector Differences (MVD) are used in bypass mode since encrypting the signs of these introduces extensive visual distortion without compromising compression efficiency or decoding appreciably.

For more secure encryption, certain schemes also incorporate Intra Prediction Modes (IPM) of the normal mode, which adds marginally to bitstream size but improves distortion resilience. Other methods extend encryption to features like luma/chroma IPM, reference frame indices, and SAO parameters, but exclude parameters such as Delta QP for ensuring codec stability. In our scheme, encryption is region of interest (ROI)-based and aimed at syntax elements correlated to sensitive spatial areas.

Step 2: Encryption Key Generation. After selecting the target elements, a master stream is encrypted with a cipher of cryptography. As each Coding Tree Unit (CTU) in HEVC is coded independently, stream ciphers such as AES (in CTR or CFB modes), RC6, or chaotic map-based algorithms would be best. The resulting key enables secure and synchronized encryption and decryption at encoder and decoder.

Step 3: Bitstream Encryption. Last but not least, the resultant bitstream is encrypted right after entropy coding. The hidden syntax components are modified using the derived key typically by XOR operations for binary and Linear Congruential (LC) functions for non binary. More sophisticated schemes scramble values of MVD and QTC as well as this to give more visual obfuscation without destroying codec compliance.
**Algorithm 3** Selective decryption scheme**Require:** 
Encrypted bitstream, secret key *K*, ROI map**Ensure:** 
Reconstructed video 1:Initialise CABAC decoder 2:Regenerate ROI masks 3:**for** each Coding Tree Unit (CTU) **do** 4:    Identify encrypted syntax elements 5:    **for** each encrypted syntax element **do** 6:        Obtain current Most Probable Symbol (MPS) 7:        **for** each encrypted bin $C_{i}$ **do** 8:           $S_{i}\leftarrow {\mathrm{AES}}_{128}\left(C_{i-1},K\right)$ 9:           ${\tilde{P}}_{i}\leftarrow S_{i}⊕C_{i}$10:           $P_{i}\leftarrow {\tilde{P}}_{i}⊕\mathrm{MPS}$11:           Send $P_{i}$ to arithmetic decoder12:        **end for**13:        **if** syntax element is non-binary (QTC or MVD) **then**14:           Apply inverse Linear Congruential function15:           $X_{i}\leftarrow a^{-1}\left(X_{i}^{\prime }-b\right)\;\mathrm{mod}\;m$16:        **end if**17:    **end for**18:**end for**19:Perform standard inverse transform and prediction20:**return** Reconstructed video

**Figure 2:**
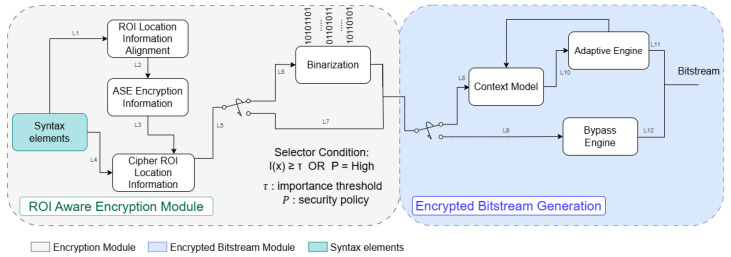
The major pipeline for CABAC coding encryption. Line definitions: L1: syntax element input; L2: aligned ROI location information; L3: encryption control metadata; L4: direct syntax element forwarding; L5: selector input; L6: encrypted path for I(x)≥τ or high security policy; L7: bypass path; L8: context coded bins; L9: bypass bins; L10: context probabilities; L11–L12: arithmetic coded and bypass coded bitstream outputs.

After these operations, the encrypted bitstream is saved or transported securely. During decoding time, a Homomorphic Decryption Decoder (HDD) recreates the key stream, decrypts the syntax elements, and undergoes regular decoding processes such as inverse transform and prediction to exactly rebuild the original video. Figure 2 demonstrates the major pipeline for CABAC encryption implementation.

The most important advantage of SE over HEVC is that selective encryption (SE) offers a very efficient way of protecting video data without making it any less compatible with the HEVC standard. An encrypted video is still decodable by a standard HEVC decoder because SE leaves the format of the encoded bitstream unchanged. This is one of the most important strengths of SE and makes SE very flexible and easy to combine with other content protection schemes such as watermarking or information hiding.

Low Bit Rate Impact: If encryption is applied to syntax elements in bypass mode, there is no effect on bit rate that can be perceived. Even when encrypting regular mode elements, for instance, the Quantized Transform Coefficients (QTC), the bit rate increase is generally less than 0.1%, and in most practical applications, this is negligible.

Visual Protection: SE effectively obscures important visual data through selectively encrypting features such as QTC, Motion Vector Differences (MVD), and Intra Prediction Modes (IPM). This distortion obscures important texture and motion information, providing an effective layer of privacy protection for sensitive visual data.

Efficiency and High Speed: Since the encryption is performed before the CABAC process and involves lightweight stream ciphers, SE enjoys high processing speed. The additional computational overhead is negligible around 1.5–2% and hence the method can be used for real-time systems as well as video’s large-scale applications.

To quantitatively analyze the interaction between selective encryption (SE) and CABAC, we evaluated the bitrate variation introduced by encrypting different classes of syntax elements under both regular and bypass coding modes. Since CABAC employs adaptive probability modeling in regular mode, encryption applied to these elements may slightly affect context adaptation, whereas bypass-coded elements are encoded assuming uniform probability and are therefore insensitive to encryption.

In the proposed framework, encryption is primarily applied to bypass-coded syntax elements such as Quantized Transform Coefficients (QTC) and Motion Vector Differences (MVD). As a result, the CABAC probability model remains unchanged, and no observable impact on compression efficiency is introduced. When encryption is extended to a limited set of regular-mode elements, such as selected Intra Prediction Modes (IPM), only minor perturbations in context modeling are observed, leading to a negligible bitrate increase.

Table 1 reports the bitrate overhead introduced by the proposed SE scheme under Random Access (RA), Low Delay (LD), and All Intra (AI) configurations. Across all tested sequence classes and coding scenarios, the bitrate increase consistently remains below 0.1%. These results confirm that the proposed selective encryption strategy introduces negligible impact on compression efficiency while preserving CABAC compatibility.

### 3.7. Choice of Encryption Algorithm

AES in counter (CTR) mode is employed in the proposed framework due to its low latency, stream-oriented operation, and suitability for real-time video coding. AES-CTR enables encryption through simple XOR operations and exhibits predictable computational complexity. In addition, AES benefits from widespread hardware support and efficient implementations on both general-purpose processors and embedded platforms.

In contrast, chaotic map-based encryption schemes typically rely on iterative and floating point computations, which introduce higher latency and complicate hardware implementation. These characteristics limit their applicability in real-time CABAC-based video compression pipelines. Therefore, AES-CTR provides a practical balance between security, efficiency, and hardware feasibility. Overall, the proposed selective encryption framework ensures effective content protection while maintaining CABAC coding efficiency, format compliance, and low computational overhead.

As shown in Table 2, the proposed method substantially reduces the time needed for privacy-sensitive pixel extraction compared with DeepSVC [49] across all tested sequences. This efficiency improvement is especially notable for high-motion content such as Football and Surfing, where the proposed approach processes regions in a fraction of the time required by DeepSVC. In our framework, sensitive regions within video frames are automatically identified using object detection models, enabling dynamic recognition and tracking of privacy-sensitive content such as faces or license plates. This automated approach eliminates the need for manual intervention and ensures that selective encryption adapts to scene changes and moving objects.

By encrypting only the detected regions, computational overhead is significantly reduced compared with full-frame encryption, while preserving visual privacy. The system is also designed to manage occlusions and multiple moving targets, maintaining consistent privacy protection in complex or dynamic environments.

### 3.8. Fully Encrypted Layer

For scenarios where there is a need for increased security, such as video surveillance or other sensitive applications, the fully encrypted layer is activated. The video stream itself is initially divided into three second segments. A hash and a Message Authentication Code (MAC) are created for each segment using the HMAC algorithm. This is a security measure so that each segment can be independently verified for integrity and authenticity throughout transmission or storage.

In order to verify the video segments, SHA-256 hash is initially determined for each segment and then an HMAC key is generated with a Message Authentication Code (MAC) using an HMAC algorithm and a randomly chosen key. Each video segment has an HMAC key, which is kept confidential and intact. At the block key generation stage, a block level HMAC key is calculated by performing randomized hashing on the HMAC key of the segment. Results from both the data encryption and key generation processes are then cached in the blockchain and buffered. Each blockchain block links to later and earlier blocks and contains information such as the video segment’s path, the Video Integrity Code of the segment (VIC), an ephemeral ECC public key, a timestamp, and the HMAC of the previous block.

**Figure 3:**
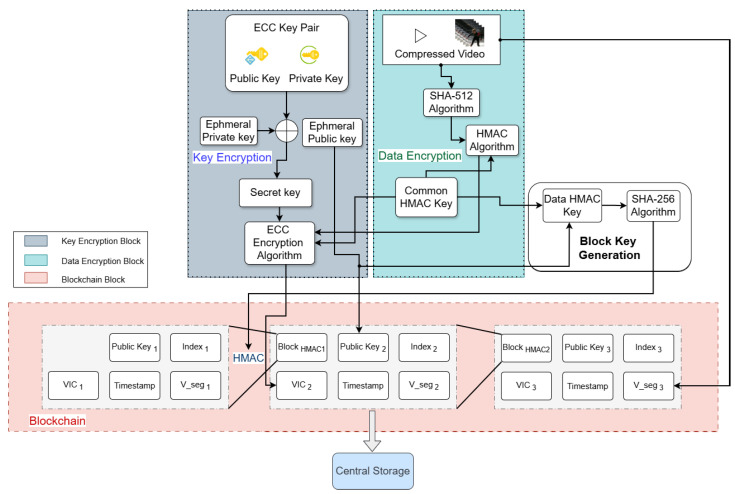
The proposed fully encrypted algorithm SHMC.

The main encryption process uses elliptic curve cryptography (ECC) to encrypt the HMAC key and MAC of the video to offer additional security and protection from illegal tampering as shown in Figure 3. The resulting process is referred to as the Fully Encrypted Video Integrity Code (FVIC) in the proposed encoder. In elliptic curve cryptography (ECC), mathematical operations are executed over finite fields, which may be categorized as either prime or binary fields. Such fields are denoted by GF(q), where $q=p^{m}$, with *p* representing a prime number and *m* a positive integer.

When m=1 and *p* is an odd prime, the field is referred to as a *prime finite field*, written as GF(p). On the other hand, if p=2 and m>1, the structure forms a *binary finite field*, or equivalently, a characteristic two field, expressed as GF$\left(2^{m}\right)$.

For fields of characteristic neither two nor three, the elliptic curve can be represented as the reduced Weierstrass form:

(5) $$y^{2}=x^{3}+ax+b$$

The discriminant:

(6) $$\Delta =-16(4a^{3}+27b^{2})$$

has to differ from 0 in order to define a valid curve. Over a finite field, the elliptic curve is given by

(7) $$y^{2}\equiv x^{3}+bx+a\;\left(\mathrm{mod}\;p\right)$$

where ≡ represents modular equivalence.

**Figure 4:**
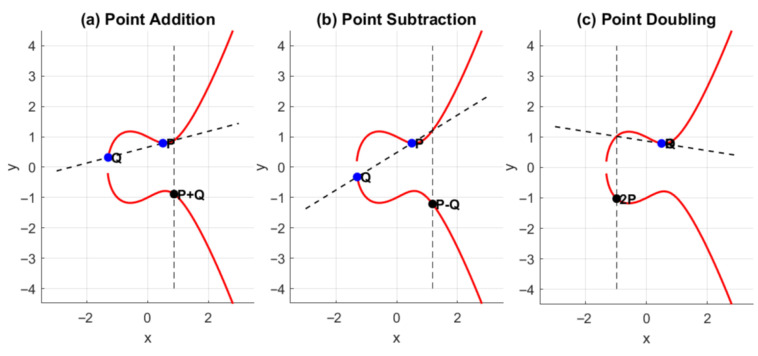
Overview of the ECC operations: (**a**) point addition, (**b**) point subtraction, and (**c**) point doubling.

Every elliptic curve is associated with parameters (p,a,b,G,n,h), where *G* is the generator point, *n* its order, and *h* the cofactor. ECC Operations and Secure Communication Figure 4 shows the main ECC operations point addition, subtraction, and doubling. For safe communication between two parties, *A* and *B*, each selects a common curve and generator point *G*. Their private keys, $K_{A}$ and $K_{B}$, lead to public keys $P_{A}=K_{A}G$ and $P_{B}=K_{B}G$, respectively.

For message $P_{m}$, sender *A* performs encryption with *B*’s public key:

(8) $$C=\left(kG,\;P_{m}⊕kP_{B}\right)$$

where *k* is randomly chosen for each encryption.

*B* recovers the original message by computing the following:

(9) $$P_{m}=\left(P_{m}⊕kP_{B}\right)⊕kP_{B}$$

Since a new random *k* is selected with each encryption, even identical messages yield different ciphertexts, which increases the security of the scheme against replay and pattern attacks.

#### ECIES Integration

For further security of keys, the suggested model adopts the Elliptic Curve Integrated Encryption Scheme (ECIES) [50], which integrates ECC-based public key cryptography with symmetric encryption for efficiency considerations. The key relationship is given as

(10) $$K_{b}=K_{v}G$$

where $K_{v}$ and $K_{b}$ are the private key and the public key, respectively. For every encryption session, a temporary key pair is generated as follows:

(11) $$k_{ue}=k_{e}G$$

This renders every encryption session unique, immune to interception, and secure in case of compromised previous session keys.

### 3.9. Computational Overhead of Blockchain-Based Verification

The fully encrypted layer incorporates blockchain-based verification using ephemeral elliptic curve cryptography (ECC) keys and HMAC authentication at the segment level. To evaluate the computational overhead introduced by this design, we analyze the additional processing cost incurred during encryption, verification, and playback, and compare it with conventional digital rights management (DRM) mechanisms.

In the proposed framework, ephemeral ECC key generation and HMAC computation are performed once per video segment rather than per frame. As a result, the cryptographic overhead scales linearly with the number of segments and remains independent of the video resolution or frame rate. Compared to traditional DRM systems that rely on persistent key exchanges or license server interactions, the proposed approach avoids repeated handshake operations during playback.

Experimental evaluation as presented in Table 3 shows that the additional processing time introduced by ECC key derivation and HMAC verification accounts for a small fraction of the overall encoding and decoding time. In practice, the average overhead remains within a few milliseconds per segment, which is negligible relative to segment duration and does not affect real-time decoding performance. These results indicate that the proposed verification mechanism achieves improved security with acceptable computational cost.

## 4. Experiment Configurations

To comprehensively evaluate the proposed framework, experiments are conducted on datasets representing diverse application domains. Surveillance-oriented sequences include static and dynamic scenes with human activities and occlusions, enabling evaluation of privacy preservation and visual distortion. Video conferencing sequences emphasize facial regions and low-latency requirements, while autonomous driving sequences contain complex motion, object interactions, and machine vision tasks such as object detection and tracking.

All datasets are encoded using common test conditions with resolutions ranging from 720p to 1080p and frame rates between 30 and 60 fps, ensuring fair and reproducible comparisons across scenarios.

We evaluate our proposed end-to-end method for the four tasks in machine vision, focusing on video object detection and action recognition. For object detection, experiments are conducted on the ImageNet VID dataset, which includes 3862 training video snippets and 555 validation videos. For action recognition, we evaluate on UCF-101 [51], where videos are organized into 25 groups, each containing 4–7 clips of a specific action; clips within the same group may share features like similar backgrounds, viewpoints, or action categories. Additionally, we use HMDB-51, consisting of 6766 clips spanning 51 action categories. Our training and evaluation procedures follow the standard protocols established by MMTracking and MMAction2.

To evaluate the effectiveness of our framework for human viewing, the structure and texture modules are trained on the Vimeo-90k dataset. Video reconstruction performance is then assessed using the HEVC common test conditions, covering different classes using sequences corresponding to UrbanScenes, CityHighway, SuburbStreets, CampusWalk, ParkTrails, MountainStreams, MixedScenesAvg, and IndoorMotion. For tasks involving both object detection and frame reconstruction, datasets containing raw (uncompressed) videos along with corresponding object annotations are required. Currently, only two datasets meet these requirements: SFU HW Objects v1 [52] and TVD [53], both of which have been used within the MPEG-VCM standardization efforts [54]. The SFU HW Objects v1 dataset consists of raw YUV420 video sequences and has previously played a role in the development of video coding standards, including HEVC [16] and VVC [18].

The proposed SHMC framework was implemented on an NVIDIA RTX 2080Ti GPU. Experimental results show that our approach surpasses existing state-of-the-art methods in detecting a variety of tampering types including copy, move, insertion, and deletion attacks while maintaining high accuracy and robustness in verifying video.

## 5. Evaluation Metrics

Following the approach adopted in many recent studies [1,35,37,43,55], we evaluate the proposed approach performance using the following criterias: For video reconstruction, we report the bitrate in bits per pixel (bpp) and assess quality through RGB peak signal to noise ratio (PSNR) and RGB multi-scale structural similarity (MS-SSIM) [43]. In terms of object detection, we use mean average precision (mAP), a common benchmark. To calculate mAP, the average precision (AP) is determined for each class as the area under its precision recall curve. Precision measures the proportion of detections that are correct, whereas recall represents the proportion of true objects successfully identified. The final mAP value is obtained by averaging the APs across all classes, producing a single metric that reflects overall detection performance.

To evaluate the effectiveness of the proposed adaptive intrusion detection system, we conducted BD-rate reduction experiments under various intrusion scenarios. Five different types of intrusions were simulated on the user interface under two baseline conditions: without selective intrusion and with full intrusion. The adaptive system was then applied, dynamically adjusting its operation based on the application context and the current intrusion situation.

**Figure 5:**
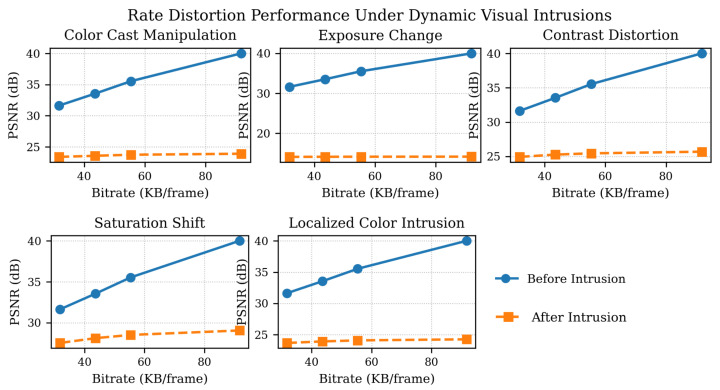
BD-rate reduction comparison for intrusions detection scenarios in the intrusion unit using the same big buck bunny HEVC test sequence [48].

The results show that the adaptive approach substantially reduces BD-rate compared to both baseline conditions, demonstrating enhanced efficiency and responsiveness in handling intrusions. This improvement underscores the benefits of context-aware adaptation in minimizing the impact of intrusions on system performance, as illustrated in Figure 5.

### 5.1. The Human Vision Applications Selection

Recent work on video understanding and multi-modal analysis has explored the fusion of visual and auxiliary features to improve machine interpretation accuracy. In addition, integration of system components in secure multimedia pipelines has been explored in broader engineering contexts [32,56].

**Figure 6:**
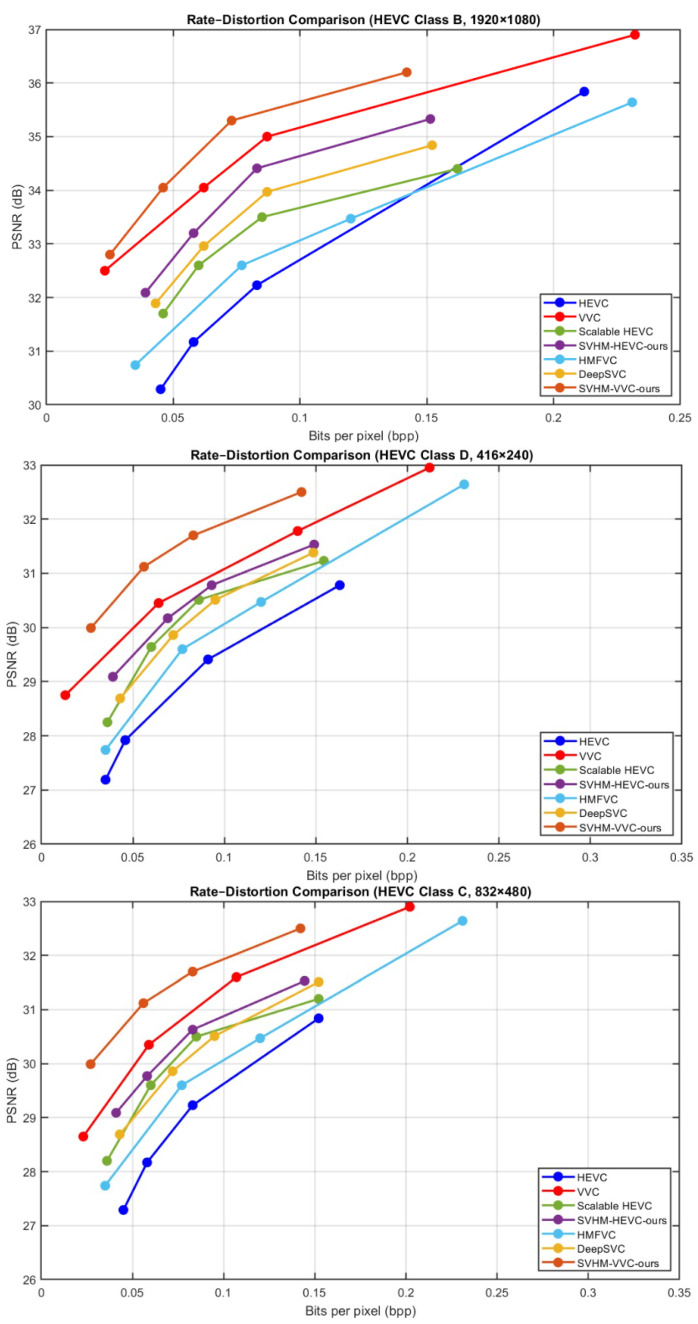
BD-rate reduction evaluation of SHMC on texture and structure layers. PSNR and the corresponding bits per pixel (bpp) are shown for different methods on HEVC common test sequences.

Figure 6 illustrates the BD-rate reduction results for SHMC, evaluated on both texture and structure layers in terms of rate-distortion (RD) performance. The PSNR values along with the corresponding bits per pixel (bpp) for different methods are plotted over the HEVC common test sequences. It is clear that SHMC consistently achieves significant BD-rate reductions compared to other methods, demonstrating improved compression efficiency while maintaining high visual quality.

These gains are observed in both the texture and structure layers, underscoring the framework’s ability to preserve fine details as well as structural information. Overall, the RD curves indicate that SHMC strikes an effective balance between bitrate and reconstruction quality, thus making it a reliable and cost effective technique for scalable video coding applications.

**Figure 7:**
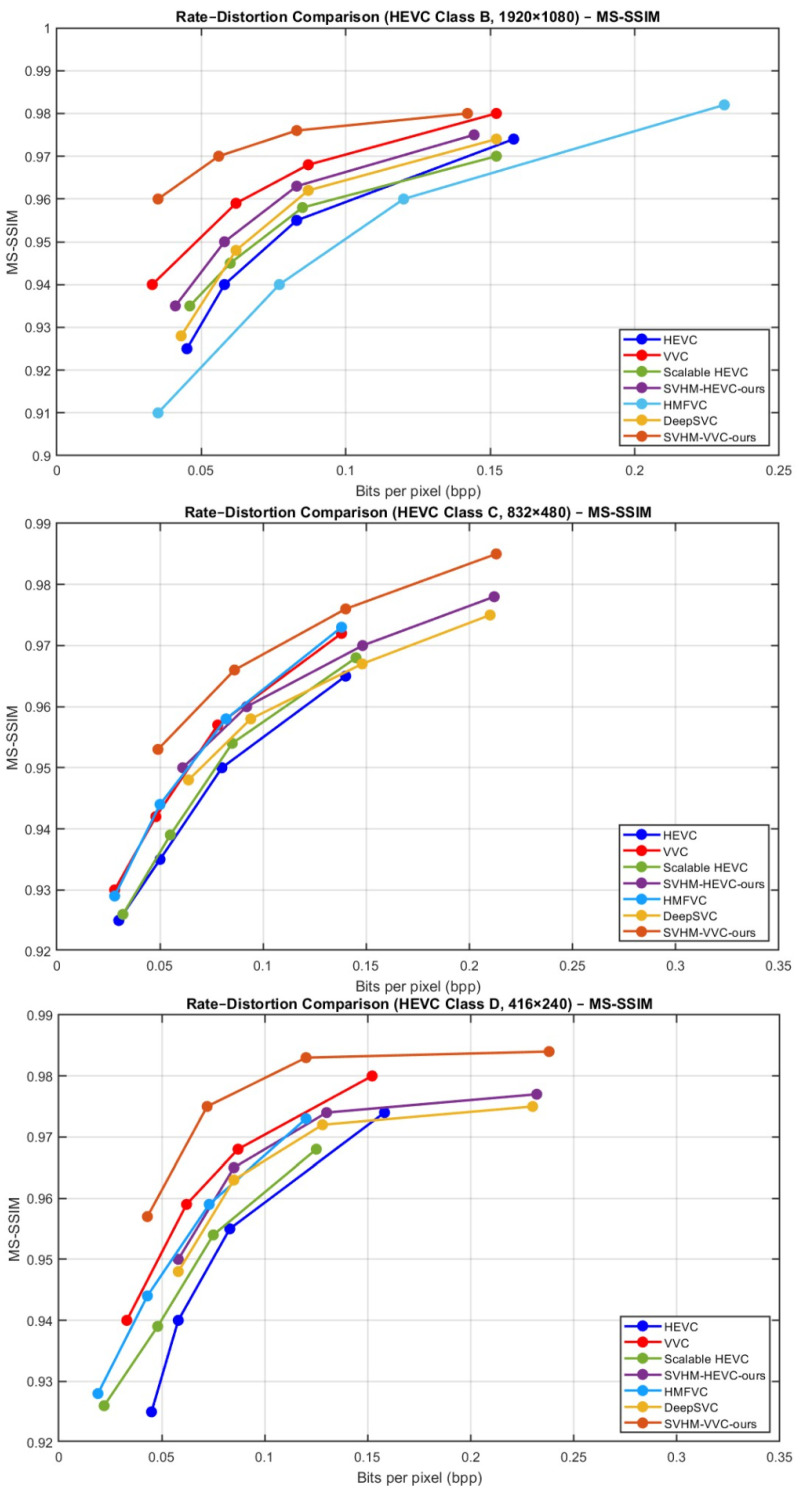
MS-SSIM RD performance of SHMC on texture and structure layers, showing MS-SSIM values versus bits per pixel (bpp) for different methods on HEVC common test sequences.

Figure 7 shows the MS-SSIM performance of SHMC on both the structure and texture layers, presented through rate distortion (RD) curves. The plots show MS-SSIM values along with the corresponding bits per pixel (bpp) for various methods applied to the HEVC common test sequences.

The results indicate that SHMC consistently outperforms other approaches, achieving higher MS-SSIM values at comparable or even lower bitrates. This demonstrates the proposed approach to preserve perceptual quality more efficiently. Performance improvements are evident across both texture and structure layers, ensuring that SHMC maintains both visual fidelity and structural detail. Overall, the RD curves highlight the effectiveness of SHMC in balancing compression efficiency with high-quality visual reconstruction in scalable video coding scenarios.

**Figure 8:**
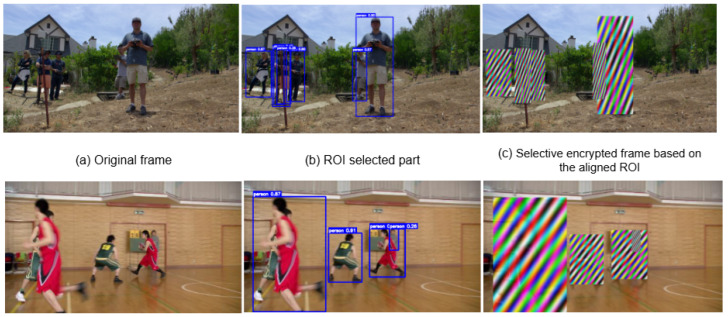
Subjective visual results for Netflix Aerial and BasketballPass-based selective ROI encryption (**a**) original frame, (**b**) ROI-selected part, and (**c**) selective encrypted frame based on the aligned ROI [48].

As shown in Figure 8, the proposed selective encryption is illustrated using the BasketballPass sequence, where the visual impact of encrypting regions of interest (ROI) can be clearly observed.

### 5.2. The Machine Analysis Applications Selection

The BD-rate performance of the proposed method in comparison with conventional and learning-based codecs across multiple video collections under the random-access configuration is presented in Table 4. The proposed method consistently achieves the largest BD-rate reductions across all datasets, indicating superior compression efficiency relative to both traditional codecs (VP9 [57], HEVC [16], and VVC [18]) and existing learning-based approaches. The performance evaluation of the proposed SHMC in video action recognition, measured on the UCF101 and HMDB51 datasets.

Table 5 summarizes the performance of various video coding methods, our proposed method across HEVC Classes B, C, and D. The results are reported in terms of BDBR measured on both PSNR and MS-SSIM versus bpp curves.

Our proposed method consistently achieves superior compression efficiency, with lower average BDBR values of −30.76% (PSNR) and −60.29% (MS-SSIM) across all classes. For Class B, our approach reduces the bit rate while maintaining high fidelity, outperforming most prior methods. Similarly, in Class C and Class D sequences, the proposed method demonstrates significant gains, particularly in challenging low-resolution Class D videos, indicating robustness across different content types. Overall, the results confirm that our method not only improves rate distortion performance over conventional codecs such as HEVC and VVC but also surpasses state-of-the-art learned video compression techniques like DeepSVC.

Table 6 presents Top-1 and Top-5 accuracy (%) along with the corresponding bits per pixel (bpp) for different methods, and our proposed method consistently achieves the highest Top-1 accuracy, reaching 79.51% on UCF101 and 42.97% on HMDB51, while maintaining the lowest bpp compared to other methods. This demonstrates that SHMC provides superior semantic layer representation, delivering higher action recognition accuracy with greater compression efficiency.

### 5.3. Selective Encryption Applications

In this part, we illustrate the experimental evaluation of the proposed selective encryption scheme for video streaming. The experiments are designed to assess both the effectiveness of encryption in protecting sensitive regions and the visual quality of the unencrypted areas. We focus on the selective encryption of regions of interest (ROI) in video sequences, demonstrating how our approach can securely conceal critical content while preserving the perceptual integrity of the surrounding scene. To illustrate the practical performance, we provide subjective visual results on the BasketballPass sequence, highlighting how the proposed method effectively encrypts only the ROIs while leaving other regions visually intact. Both subjective visual results and objective metrics are provided to demonstrate the advantages of our approach in terms of security, efficiency, and visual fidelity.

Figure 8 illustrates the subjective visual results of the BasketballPass sequence under selective ROI encryption. This selective encryption preserves the overall scene context, ensuring that non-sensitive areas remain perceptible, while the targeted regions are securely protected. The results clearly demonstrate that our approach achieves high visual security in the ROI without introducing noticeable artifacts or degrading the quality of the unencrypted regions, confirming the effectiveness and practicality of the proposed selective encryption scheme.

### 5.4. Intrusion Scenarios

A quantitative evaluation of the impact of intrusion scenarios on video quality under compression was performed, focusing on their combined effect on both human visual perception and machine-oriented analysis. The performance of the intrusion system is assessed by comparing rate distortion behavior before and after intrusion across multiple compression settings, using PSNR as an objective quality metric referenced to the original clean frames. The five selected scenarios are as follows.

#### 5.4.1. Brightness Shift Intrusion

Brightness shift intrusion refers to the intentional manipulation of video frame luminance, causing frames to appear significantly brighter or darker than their natural exposure. This type of attack alters the overall visibility of a scene, often leading to washed-out highlights or obscured shadow regions. From a security perspective, excessive brightness can hide critical details such as facial features or license plate numbers, while reduced brightness can conceal objects entirely. For machine vision systems, brightness distortion disrupts pixel intensity distributions, leading to unstable feature extraction and reduced confidence in object detection or tracking models. In practice, this attack may occur through malicious control of camera gain settings or deliberate exposure tampering, especially in outdoor surveillance systems where lighting conditions are assumed to be trustworthy.

#### 5.4.2. Contrast Distortion Intrusion

Contrast distortion intrusion involves modifying the difference between light and dark regions in a frame, either flattening the visual structure or exaggerating intensity variations. When contrast is reduced, object boundaries become less distinguishable, making it difficult for both humans and algorithms to separate foreground objects from the background. Conversely, excessive contrast can saturate details and introduce artificial edges that confuse machine perception. This type of intrusion is particularly dangerous in automated monitoring systems, as many vision models rely on contrast-driven gradients to detect shapes and motion. A realistic attack scenario includes tampering with camera processing pipelines or injecting altered video streams that subtly reduce contrast, thereby degrading detection accuracy without raising immediate suspicion.

#### 5.4.3. Color Cast Manipulation Intrusion

Color cast manipulation intrusion refers to the deliberate alteration of color balance by disproportionately amplifying or suppressing specific color channels. This results in an unnatural color tint across the entire scene, such as a strong blue, green, or yellow bias. For human observers, color cast distortion affects visual realism and can obscure important cues like skin tone or object material. For machine learning models, which often rely on color consistency for classification and recognition, this intrusion can significantly degrade performance. Such attacks are particularly harmful in systems trained under normal lighting assumptions. In real world scenarios, color cast manipulation may be introduced through white balance tampering, colored light sources, or adversarial interference in camera firmware.

#### 5.4.4. Gamma Distortion Intrusion

Gamma distortion intrusion targets the nonlinear relationship between pixel intensity and perceived brightness, reshaping how mid tone values are represented. This attack does not simply make the frame brighter or darker but redistributes intensity levels in a way that suppresses critical structural information. As a result, important visual details may appear flattened or overly enhanced, misleading human interpretation. For machine vision systems, gamma distortion disrupts learned feature hierarchies, particularly in convolutional neural networks that depend on consistent intensity patterns. A plausible attack scenario involves manipulating camera gamma correction parameters or injecting pre processed video streams that intentionally distort tonal responses, thereby reducing recognition reliability while maintaining plausible visual quality.

#### 5.4.5. Sensor Noise Injection Intrusion

Sensor noise injection intrusion simulates electronic interference or low-quality sensor behavior by introducing random intensity fluctuations into video frames. This form of attack degrades image clarity by corrupting fine details and edges, which are essential for both human interpretation and automated analysis. While humans may perceive the scene as grainy or unstable, machine learning models are especially vulnerable, as noise disrupts gradient consistency and increases false detections. Sensor noise injection can occur through electromagnetic interference, low-light manipulation, or deliberate signal corruption in the acquisition pipeline. This intrusion is particularly dangerous because it can degrade system performance gradually, making detection difficult while continuously reducing the analytical accuracy.

Figure 5 illustrates visual comparison of frames captured before and after an intrusion event in the intrusion detection scenario, demonstrating successful intrusion detection by the proposed system.

### 5.5. Fully Encryption Applications

The experimental results demonstrate the effectiveness of the proposed full encryption in real-time video streaming application. Table 7 presents a detailed comparison of the proposed method against several state-of-the-art approaches in terms of visual quality and computational performance. Metrics such as PSNR, MSE, BER, VQM, SSIM, and VMAF evaluate the reconstruction quality, while encryption and decryption times assess computational efficiency. The proposed method consistently achieves the highest PSNR (42.9 dB), lowest MSE ($10^{-9}$), and top SSIM (0.93), indicating superior fidelity. Additionally, it maintains a very low BER and high VQM and VMAF scores, demonstrating robustness and perceptual quality. In terms of efficiency, the proposed approach offers competitive encryption and decryption times compared to other methods, highlighting a favorable balance between performance and speed. Overall, the results confirm that the proposed framework outperforms existing techniques across both quality and computational metrics.

**Figure 9:**
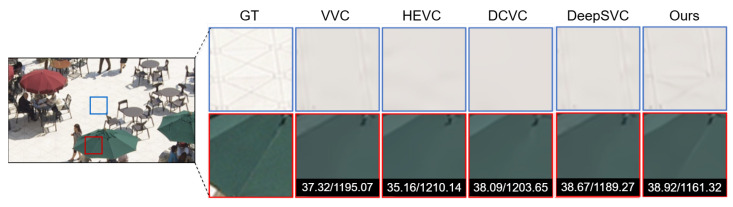
The BQSquare sequence [48] visual results comparing VVC, HEVC, DSVC, and the proposed end-to-end encryted method based on PSNR and bitrate.

**Figure 10:**
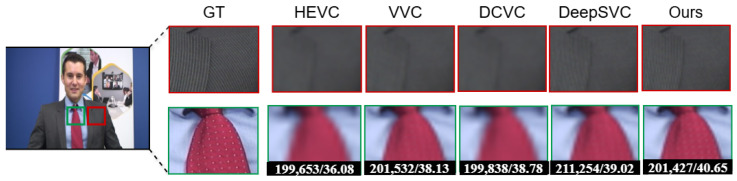
The Johnny sequence [48] visual results comparing VVC [18], HEVC [16,17], DSVC, and the proposed end-to-end encrypted method based on PSNR and bitrate.

In Figure 9 and Figure 10, the sequences test results demonstrate the comparative performance of VVC, HEVC, Deep Scalable Video Coding (DSVC), and our proposed method in terms of PSNR and bitrate. VVC achieves the highest coding efficiency, delivering superior PSNR at significantly lower bitrates compared to HEVC, which requires higher bitrates to reach similar visual quality. DSVC provides a scalable coding structure, offering competitive PSNR while enabling layer wise decoding for adaptive streaming scenarios.

Our proposed method further improves on these results using the multiframe enhancement, achieving PSNR comparable to or slightly higher than VVC while reducing the bitrate even further, highlighting its ability to maintain high visual quality at reduced bandwidth. These results confirm the effectiveness of our approach in enhancing compression efficiency and visual fidelity, making it particularly suitable for applications requiring high-quality, low-bitrate video streaming or selective layer decoding.

## 6. Conclusions

Secure high-quality reconstruction and accurate machine analysis are critical for video streaming applications. Storage and bandwidth resources are often constrained while meeting extensive surveillance demands. To effectively manage system resources, such as computing, caching, and communication, a highly efficient video codec becomes essential. This paper goes a step further by designing flexible adaptive reconstruction networks for video streaming. To address this, we introduce SHMC. On the encoder side, SHMC compresses videos into semantic, structural, and textural layers, with each representation extracted and encoded into compact, scalable bitstreams. The decoder can then selectively reconstruct partial bitstreams for semantic analysis or utilize additional layers for high quality visual reconstruction, depending on the application. In the case of intrusion detection, the encrypted layer is enabled, and following decryption, the multiframe enhancement module refines the reconstructed video stream. For machine analysis tasks, the selective encoder is employed. Experimental results demonstrate that DeepSVC consistently surpasses both conventional and learning-based codecs in terms of video reconstruction quality and machine analysis accuracy.

With this design, SHMC achieves efficient and high-quality recovery, marking the first end-to-end deep model capable of large-scale reconstruction and bringing the technology closer to real world use. Beyond surveillance and video conferencing, the proposed framework is applicable to a wide range of video-centric domains with heterogeneous requirements. In telemedicine, selective encryption can protect sensitive patient information while preserving diagnostically relevant visual features for clinical analysis. In autonomous driving, scalable representations enable efficient transmission of perception-critical regions to support object detection and tracking under bandwidth constraints. For live broadcasting and streaming applications, the framework can balance visual quality, latency, and security by adapting scalable layers to network conditions and audience requirements. These examples illustrate the flexibility of the proposed approach and its potential for deployment across diverse real-world video delivery scenarios. Despite the promising results demonstrated in this study, several open issues remain and will be addressed in future work. First, although the proposed framework shows strong performance across diverse datasets, further validation on ultra-high-resolution content (e.g., 4 K and 8 K videos) and under more stringent real-time constraints would provide deeper insights into its scalability. Second, the current implementation focuses on selected machine vision tasks; extending the framework to additional downstream applications such as video segmentation and multi-object tracking represents a valuable direction for future study. SHMC is not tied to a specific backbone; different deep models can be integrated into the framework. Another open direction is enabling fast adaptation to varying compression ratios, which will be explored in future work. Beyond surveillance and video conferencing, the proposed framework is applicable to a wide range of video-centric domains with heterogeneous requirements. In telemedicine, selective encryption can protect sensitive patient information while preserving diagnostically relevant visual features for clinical analysis. In autonomous driving, scalable representations enable efficient transmission of perception-critical regions to support object detection and tracking under bandwidth constraints. For live broadcasting and streaming applications, the framework can balance visual quality, latency, and security by adapting scalable layers to network conditions and audience requirements. These examples illustrate the flexibility of the proposed approach and its potential for deployment across diverse real-world video delivery scenarios.

## Figures and Tables

**Table 1:** Bitrate overhead introduced by selective encryption.

Sequence Class	RA (%)	LD (%)	AI (%)
Class B	0.03	0.05	0.04
Class C	0.02	0.04	0.03
Class D	0.01	0.03	0.02
Class E	0.04	0.06	0.05
Average	0.025	0.045	0.035

**Table 2:** Time comparison for extracting pixels in privacy-sensitive regions.

Sequence	DeepSVC (s) [49]	Proposed (s)
Baseball	39.08	22.67
Football	36.52	0.0032
Horse	21.49	1.83
Surfing	7.36	0.00264
Elephant	5.43	0.0421
Vase	4.74	0.18

**Table 3:** Computational overhead of the fully encrypted layer compared with conventional DRM mechanisms.

Method	Key/Segment	Verification Time (ms)	Playback Impact
Conventional DRM	Persistent	12.84	Medium
Chaotic Map Encryption	Per frame	6.48	High
Proposed ECC + HMAC (Segment-level)	Ephemeral	2.01	Negligible

**Table 4:** BD-rate (%) comparison across codecs and video collections. Lower is better using random access configuration.

Dataset	VP9	HEVC	VVC	Learned-DCVC	DCVC-TCM	DCVC-HEM	DCVC-DC	Proposed Method
UrbanScenes	54.81	40.82	0.00	168.37	0.27	−19.74	−35.06	−38.76
CityHighway	48.77	36.67	0.00	85.81	−14.66	−27.74	−39.67	−45.48
SuburbStreets	65.06	44.72	0.00	131.66	−3.56	−18.49	−32.07	−34.79
CampusWalk	40.99	30.76	0.00	162.16	33.04	11.53	−24.07	−30.07
ParkTrails	39.08	29.83	0.00	136.30	12.97	−1.97	−37.01	−42.56
MountainStreams	67.05	45.01	0.00	285.24	13.43	1.15	−33.68	−32.52
MixedScenesAvg	46.69	35.65	0.00	146.61	10.21	−6.38	−34.28	−38.19
IndoorMotion	57.74	42.94	0.00	139.63	10.40	−13.82	−29.35	−29.67

**Table 5:** Evaluation of the proposed HMSVC texture layer using BD-rate (%) metrics. Performance is assessed based on PSNR per bit and MS-SSIM per bit metrics. Red highlights the top-performing method, while Blue marks the second-best result.

Dataset	HEVC [16]	VVC [18]	TMM2025 [26]	TMM25 [37]	TPAMI2024 [41]	TIP2024 [58]	TB [59]	TMC2024 [60]	DeepSVC [49]	Proposed Method
Class B	58.12/58.74	12.91/9.17	30.59/7.57	−23.94/−58.87	69.04/−13.06	−20.46/−57.38	3.63/−41.80	−30.17/−59.85	−26.88/−62.38	−27.21/−62.43
Class C	34.15/31.47	8.35/4.77	48.67/−0.66	3.71/−50.35	59.32/−19.62	−7.06/−45.04	−25.95/−51.33	5.93/−44.00	−22.56/−54.87	−26.39/−54.79
Class D	15.48/17.81	−4.48/−2.27	32.53/−13.90	−17.05/−58.62	21.71/−25.26	−25.04/−60.17	−1.73/−44.28	−34.52/−60.01	−33.45/−62.35	−38.67/−63.65
Average	35.92/36.01	5.59/3.89	37.26/−2.33	−12.42/−55.95	50.02/−19.31	−17.52/−54.20	−8.01/−45.80	−19.59/−54.62	−27.63/ −59.87	−30.76/ −60.29

**Table 6:** Evaluation of machine analysis for video action recognition. The semantic layer is assessed using Top-1 and Top-5 accuracy (%) along with corresponding bpp for different methods on UCF101 and HMDB51.

Dataset	DCVC [61]	HEVC [16]	VVC [18]	SHVC [17]	DeepSVC [49]	Ours
UCF-101	57.96/80.84/0.1527	74.25/91.65/0.0409	76.02/93.55/0.0441	76.63/93.87/0.0337	79.49/93.60/0.0316	79.51/93.62/0.0315
HMDB-51	33.01/59.87/0.0378	39.35/68.69/0.0369	40.65/70.65/0.0418	40.46/69.80/0.0282	42.94/72.88/0.0279	42.97/72.91/0.0281

**Table 7:** Comparative analysis of encryption-integrated video frameworks, including ECC-Guard [50], DeepKeyNet [62], 3D-VEncrypt [45], VisSecure-CNN [55], and BiLSTM-VidEnc [63]. The proposed AES-BlockAdapt model achieves the best balance between video quality and computational overhead.

Model	PSNR	MSE	BER	VQM	SSIM	Enc. Time (s)	Dec. Time (s)	VMAF
AES-BlockAdapt (Proposed)	42.9	0.22	$10^{-9}$	98.4	0.93	4.64	0.10	97.1
ECC-Guard	42.5	0.25	$10^{-9}$	98.3	0.92	4.49	0.11	96.5
DeepKeyNet	31.6	0.40	$10^{-7}$	88.5	0.86	5.62	0.28	92.3
3D-VEncrypt	29.3	0.36	$10^{-5}$	88.9	0.73	6.09	0.35	92.7
VisSecure-CNN	17.8	0.49	$10^{-4}$	91.6	0.65	7.39	0.45	89.6
BiLSTM-VidEnc	20.5	0.57	$10^{-2}$	92.3	0.43	8.90	0.45	90.7

## Data Availability

The datasets used in this study are publicly available and can be accessed from the original sources cited in the manuscript.
